# HN1L promotes migration and invasion of breast cancer by up‐regulating the expression of HMGB1

**DOI:** 10.1111/jcmm.16090

**Published:** 2020-11-16

**Authors:** Dechuang Jiao, Jingyang Zhang, Ping Chen, Xuhui Guo, Jianghua Qiao, Jiujun Zhu, Lina Wang, Zhenduo Lu, Zhenzhen Liu

**Affiliations:** ^1^ Department of Breast Disease Henan Breast Cancer Center Affiliated Cancer Hospital of Zhengzhou University & Henan Cancer Hospital Zhengzhou China; ^2^ College of Basic Medical Sciences Collaborative Innovation Center of Henan Province for Cancer Chemoprevention Zhengzhou University Zhengzhou China

**Keywords:** breast cancer, HMGB1, HN1L, HSPA9, invasion and metastasis

## Abstract

Recent reports showed that haematological and neurological expressed 1‐like (HN1L) gene participated in tumorigenesis and tumour invasion. However, the expression and role of HN1L in breast cancer remain to be investigated. Here, bioinformatics, western blot and immunohistochemistry were used to detect the expression of HN1L in breast cancer. Wound healing, transwell assay, immunofluorescence assay and mass spectrum were used to explore the role and mechanism of HN1L on the migration and invasion of breast cancer, which was confirmed in vivo using a nude mice model. Results showed that HN1L was significantly over‐expressed in breast cancer tissues, which was positively correlated with M metastasis of breast cancer patients. Silencing HN1L significantly inhibited the invasion and metastasis of breast cancer cells in vitro and lung metastasis in nude mice metastasis model of breast cancer. Mechanistically, HN1L interacted with HSPA9 and affected the expression of HMGB1, playing a key role in promoting the invasion and metastasis of breast cancer cell. These results suggested that HN1L was an appealing drug target for breast cancer.

## 1 INTRODUCTION

Breast cancer is the most common cancer in women worldwide.^1^, ^2^ The common sites for metastatic spread are bone, lung and liver.^3^, ^4^ Usually, the 5‐year survival rate of advanced breast cancer is only 20%.^5^ Metastasis and recurrence have become major obstacles to the survival of breast cancer patients. Therefore, screening and identification of the regulatory molecules closely related to the invasion and metastasis and exploring the molecular mechanisms are of great significance for the diagnosis and treatment of breast cancer.

Haematological and neurological expressed 1‐like (HN1L) belongs to the haematological and neurological expressed 1 (HN1) family.^6^ The human HN1L gene, also known as L11, encodes a 190‐aa protein. Previous reports showed that the expression of HN1 was up‐regulated in prostate adenocarcinomas.^7^, ^8^ Moreover, HN1 could distinguish human ovarian carcinoma from healthy ovarian epithelial tissue.^9^ Besides, HN1 negatively influenced the β‐catenin/E‐cadherin interaction, and contributed to migration in prostate cells.^10^ HN1 also contributed to the migration, invasion and tumorigenesis of breast cancer by enhancing MYC activity.^11^ However, not much is known about HN1L cellular function. In 293T cells, HN1L mainly activates cell cycle‐related signalling pathways such as E2F, Rb and P53. Over‐expression of HN1L promotes cell malignant proliferation in non‐small cell lung cancer.^11^ Besides, inhibition of HN1L reduces tumour cell proliferation, cancer stem cell self‐renewal and migration, tumour growth and metastasis and is associated with improved patient survival.^12^ Here, we describe the essential role of HN1L in metastasis of breast cancer and explore its mechanism of action in this context.

We found that the expression of HN1L was up‐regulated in breast cancer tissues, and patients with high HN1L expression have a poor prognosis. Knockdown of HN1L not only inhibited the invasion and metastasis of breast cancer cells MDA‐MB‐231 and BT‐549, but also inhibited lung metastasis in nude mice metastasis model of breast cancer. Silencing HN1L effectively down‐regulated the expression of HMGB1. Moreover, the expression of HMGB1 was likely to be regulated by the HN1L/HSPA9 interaction. These findings revealed that HMGB1 may be a key protein in HN1L promoting invasion and metastasis of breast cancer and suggested that HN1L was an appealing drug target for breast cancer.

## 2 MATERIALS AND METHODS

### 2.1 Cell culture

Breast cancer cell lines MCF‐10A, MCF‐7, MDA‐MB‐231, MDA‐MB‐468, BT549, T47D were grown in DMEM medium supplemented 10% FBS. All cell lines were obtained from the American Type Culture Collection (ATCC). Cell lines were authenticated using Short Tandem Repeat (STR) analysis as described in 2012 in ANSI Standard (ASN‐0002) by the ATCC Standards Development Organization (SDO) and in Capes‐Davis et al Cell line BT549 STR profile report was received in Jul 12th 2016. Cell line MDA‐MB‐231 STR profile report was received in Oct 31th 2017.

### 2.2 Tissue samples

Fresh human tissue samples including breast cancer tissues and adjacent mammary tissues were collected from Henan Cancer Hospital. A cohort of 115 paraffin‐embedded archived breast cancer specimens were used to determine the clinical significance of HN1L, which were clinically diagnosed as breast cancer from 2013‐2014. In this study, adjacent tissues were defined as 2.0 cm from the tumour margin. Histological diagnosis and tumour‐node‐metastasis (TNM) stages of cancers were determined in accordance with the American Joint Committee on Cancer (AJCC) manual criteria breast cancer.^13^

### 2.3 HN1L expression and patient survival analysis

We selected pairs of sample data of RNA‐seq and RNAseqV2 from TCGA database for analysis. Data standardization uses the TMM (Trimmed Mean of M‐values) method. We used Mann‐Whitney *U* test to analyse the significant difference of HN1L expression at different levels in different clinical data. We used Spearman's test to analyse the correlation between the expression level of HN1L in cancer tissues and clinical data. The differential expression of the original data of HN1L in 106 pairs of TCGA RNA‐seq samples is represented by a line chart. The screening criteria for significant differences must be met | Fold change | should be greater than 2, and the *P*‐value value should be less than .05. According to the median value of HN1L expression as a cut‐off value, HNL1 was marked as high expression and low expression. Finally, the relationship between the expression of HNL1 and the overall survival of breast cancer patients was analysed by Kaplan‐Meier survival curve.

### 2.4 Immunohistochemistry (IHC)

Tissue paraffin blocks from 115 breast cancer patients were selected and sectioned for immunohistochemistry. Briefly, the tissue sections (4 μm) were dehydrated and subjected to peroxidase blocking. Primary antibodies were added and incubated at 4°C overnight in a humidified chamber, the slides were incubated with horseradish peroxidase‐conjugated secondary antibody and stained with the DAB substrate. The stained slides were observed under microscopy, and images were acquired.^14^ The percentage of HN1L‐positive cells was scored as 0, <5%; 1, 5%‐25%; 2, 25%‐50%; 3, 50%‐75%; 4, 75%‐100%. The intensity of HN1L‐positive staining was scored as 0, negative; 1, weak; 2, moderate; 3, strong. The total score was determined by the following formula: Staining index = positive percentage × intensity. The cut‐off value for HN1L level was 4, so score ≤4 was considered as low level, and score >4 was considered as high level.

### 2.5 Cell proliferation assay

Cell viability was performed by MTT assay according to the manufacturer's instructions. Briefly, approximately 3 × 10^3^ cells were seeded in 100 μL per well DMEM/RPMI 1640 in a 96‐well plate for 96 hours. Cells were then incubated with MTT (5 mg/mL) for another 4 hours at 37°C. After the medium was carefully removed, 100 μL of dimethyl sulfoxide was added and agitated to dissolve the formazan crystals. The absorbance at 490 nm was measured on a spectrophotometric plate reader. Each group was repeated in three different wells.

### 2.6 Generation of gene knockdown stable cell lines

Stable gene knockdown cell lines were generated as previously described.^15^ Briefly, the short hairpin RNA sequences of HN1L were amplified and cloned into the GV115 vector (GeneChem) between Age I and EcoR I sites for expression driven by the hU6 promoter. The positive bacterial colony was transferred to 150 mL of LB liquid medium containing Amp antibiotics and cultured overnight at 37°C shaker. The plasmid was extracted according to the EndoFree Maxi Plasmid Kit instructions (TIANGEN, DP117), and the right gene sequence plasmid was used for viral packaging. MDA‐MB‐231 and BT549 cells were plated in six‐well plate and infected with lentivirus containing shRNA. And then cells were cultured in a 5% CO_2_ incubator at 37°C for another 72 hours. Cells were harvested and total RNA and protein were extracted to determine the knockdown efficiency using real‐time PCR and western blot. The sequences of the shRNA are as follows:

shHN1L: 5′‐CTAATAGGATGGCATCTAA‐3′;
shHN1L‐2^12^: 5′‐CGCCTGTATTTGGAAGATTTAA‐3′;
shHSPA9: 5′‐ACATTGTGAAGGAGTTCAA‐3′;

The negative control was 5′‐TTCTCCGAACGTGTCACGT‐3′.

### 2.7 Construction of HMGB1‐over‐expression cell lines

The lentivirus vectors, GV358‐Flag‐HMGB1 (NM_002128) was generated by cloning the cDNA into GV358 vector (Genechem). The packaging plasmids including pHelper1.0 (15 μg), pHelper2.0 (20 μg) and the lentivirus vectors mentioned above (20 μg) were transfected into 293T cells using transfection reagent (Genechem). The media containing the retroviruses were collected 72 hours after transfection and virus were condensed and titered to 5‐8 E + 08 TU/mL. Viral transduction was performed by incubating the cells with the viruses supplemented with Polybrene (Genechem) overnight at 37°C.

### 2.8 Western blotting

Lentivirus‐infected MDA‐MB‐231 and BT549 cells were collected, lysed and centrifuged, the supernatant was collected for western blot analysis.^16^ The equal amount of protein was separated by 12% SDS‐PAGE gels and transferred onto polyvinyl‐difluoride (PVDF) membranes. Then membranes were incubated overnight at 4°C with primary antibodies, including anti‐HN1L (Abcam, NM‐144570), HMGB1 (Cell Signaling Technology, 6893S), Flag (Sigma, F1804), E‐cadherin (Cell Signaling Technology, #14472), N‐cadherin (Cell Signaling Technology, #13116), Slug (Cell Signaling Technology, #9585), Snail (Cell Signaling Technology, #3895), Vimentin (Cell Signaling Technology, #3932s), β‐catenin (Cell Signaling Technology, #9562), Twist1 (Abcam, ab50581), HSPA9 (Abcam, ab2799) and GAPDH (Santa Cruz, sc‐32233), followed by incubating with secondary antibodies labelled with horseradish peroxidase for 2 hours at room temperature. At last, proteins were detected by using the ECL plus reagents (Beyotime). Densitometric analysis for the quantification relative to GAPDH was performed using the Image J software.

### 2.9 Wound healing assay

Lentivirus‐infected MDA‐MB‐231 and BT49 cells were seeded in 6 cm cell culture dishes (1 × 10^5^ cells per dish) and cells were incubated overnight to 60%‐70% confluence cell monolayer. A wound was incised in the central area of the confluent by a 100 μL pipette tip and the dish was washed three times with PBS to remove detached cells. Add low concentration serum medium (0.5% FBS) to take photos at 0 hour 24 hours and 48 hours. The migration area was analysed by Celigo. The pictures of 0 hour, 24 hours and 48 hours after migration were obtained by Celigo scanning (the magnification of the cells was 50 times), and the cell area at 0 hour, 24 hours and 48 hours after migration was analysed. Migration area = migration cell area of 24/48 hours − migration cell area of 0 hour. According to the migration area, we judged the difference of cell healing ability between the relatively negative control group and the HN1L gene silencing group. Migration rate = (24/48 hours migration cell area − 0 hour cell migration cell area)/0 hour cell migration cell area.

### 2.10 Cell migration and invasion assays

The migration and invasion assays were performed in a 24‐well Boyden chamber with 8 μm pore size polycarbonate membrane. For migration assay, 100 μL of serum‐free medium (containing 1 × 10^5^ cells) was added to the upper compartment of the chamber, while the lower compartment was filled with 600 μL of DMEM supplemented with 30% FBS. After incubation at 37°C for 24 hours, the tumour cells remaining inside the upper chamber were removed with cotton swabs. The cells on the lower surface of the membrane were stained with 2‐3 drops of Giemsa for 3‐5 minutes after fixation with methanol. Then, soaking and washing the chamber several times and drying at room temperature. Pictures were taken under a microscope (100×, 200×). The procedures and the analyses of invasion assay were the same as those for the migration assay except for the presence of the Matrigel (Corning).

### 2.11 Co‐immunoprecipitation assay

The 3 × FLAG‐HN1L interacting complex was purified using the anti‐FLAG magnetic beads (Sigma) according to the manufacture's protocol. In brief, 3 × FLAG‐HN1L expressing cells and the control cells were lysed respectively with lysis buffer (20 mmol/L Tris‐HCl pH7.5, 150 mmol/L NaCl, 1% Triton X‐100) supplemented with protease inhibitor cocktail and phosphatase inhibitor cocktail. Equal amount of protein derived from each cell pool was incubated with anti‐FLAG magnetic beads at 4°C overnight. After immunoprecipitation, the anti‐FLAG beads were washed with TBS buffer (50 mmol/L Tris‐HCl, pH7.4, 150 mmol/L NaCl) to eliminate non‐specific binding. Immunoprecipitates were then eluted by 150 μg/mL of 3 × FLAG peptide (Sigma).

Eluates were either detected with HSPA9 and Flag antibodies for the interaction of HSPA9 with HNIL or separated by 12% SDS‐PAGE gel and stained with Coomassie Blue staining (CBB) for the LC‐MS/MS analysis.

### 2.12 LC‐MS/MS analysis

The experiments were performed on an EASY‐nLC 1000 system (Thermo Fisher Scientific) connected to an Orbitrap Fusion mass spectrometer (Thermo Fisher Scientific). The peptide was resuspended with 10 μL solvent A (water with 0.1% formic acid) and was loaded onto the trap column (Thermo Scientific Acclaim PepMap C18, 100 μm × 20 mm) and subsequently separated on the analytical column (Acclaim PepMap C18, 150 μm × 12 cm) with a flow rate of 600 nL/min for 3 minutes. Elution gradient consists of mobile phase B (99.9% acetonitrile, 0.1% formic acid) and mobile phase A. Elution gradient solutions were added as follows: B was increased from 5% to 10% in 16 minutes, 10%‐30% in 55 minutes, 30%‐95% in 1 minutes and 90% for 6 minutes.

MS data were acquired by Orbitrap Fusion Tribrid Mass Spectrometer with an ESI nanospray source.^17^ The instrument was set to run in top speed mode with 3 seconds cycles for the survey and the MS/MS scans, the spray voltage was set at 1.9 kV, the Orbitrap Fusion MS used 120 K resolving power setting for MS1 and rapid scan in ion trap analyser for MS/MS. The maximum injection time for MS was 60 ms and MS/MS was 80 ms, scan range of 400‐2000 m/z, acquisition time 78 minutes.

### 2.13 Immunofluorescence assay

Immunofluorescence assay was performed to evaluate the expression of HN1L and HSPA9. Breast cancer cells were plated on a 96‐well plate. The cells were fixed in 3.7% formaldehyde for 10 minutes and permeabilized with 0.2% Triton X‐100 in phosphate‐buffered saline (PBS). The cells were labelled with antibodies for 1 hour at room temperature, rinsed in PBS and incubated with a fluorescein isothiocyanate‐conjugated secondary antibody for 45 minutes at room temperature. The nuclei were stained with DAPI for 10 minutes. Finally, cells were visualized using immunofluorescence microscope and photographed with a camera.

### 2.14 RNA extraction and quantitative real‐time PCR

Total RNA of breast cancer cells and tissues were isolated using TRIzol Reagent (Invitrogen), and the first strand cDNA was synthesized with TransScript Reverse (TransGen Biotech). The cDNA was quantified by real‐time quantitative PCR using SYBR Green Real‐Time PCR Master Mixes (Applied Biosystems) and a Real‐time GAPDH was used as an internal control. Primers are as follows: HN1L: forward, 5′‐GGTATCTTTGACGAATCAACCCC‐3′; reverse, 3′‐CAGTGACCGGAGACCCAAAAA‐5′; HSPA9: forward, 5′‐GGAAGGTAAACAAGCAAAGGTGC‐3′; reverse, 3′‐CCAACAAGTCGCTCACCATCT‐5′; GAPDH: forward, 5′‐TGACTTCAACAGCGACACCCA‐3′; reverse, 3′‐CACCCTGTTGCTGTAGCCAAA‐5′;

### 2.15 In vivo animal experiments

Tumour formation assay was done according to previous reports.^18^ Small living animal imaging technology was used to detect the occurrence of tumour metastasis.^19^ BALB/c nude mice were purchased from Shanghai Ling Chang Biological Technology Co., Ltd (SCXK2013‐0018). All animal experiments were performed under the approval of Institutional Animal Care and Use Committee (IACUC) at Henan Cancer Hospital (Permit No: 2014ct001). Logarithmic growth of breast cancer cells and HN1L knockdown MDA‐MB‐231 cells were prepared and (2 × 10^7^/mL) injected into the tail vein slowly.^20^ A live imaging test was performed once a week. The tumour volumes were calculated as tumour volume = Length × Width^2^/2.^21^ Tumour size was determined by caliper measurement. Tumour tissues were harvested, photographed and weighted.

### 2.16 GeneChip prime view

The total RNA of the samples (Control vs shHN1L) were extracted by Trizol method, and the total RNA was tested by NanoDrop 2000 and Agilent Bioanalyzer 2100, and the qualified samples were put into the chip experiment. First, the cDNA, was synthesized by one strand, and then the double DNA template was synthesized by two strands, and then the biotin‐labelled aRNA (amplified RNA) was obtained by inversion in vitro. The aRNA was purified and then fragmented and hybridized with the chip probe. After the completion of hybridization, the chip was washed and dyed, and finally scanned to get the picture and the original data.

### 2.17 Ethical approval and consent to participate

All animal procedures and care were conducted in accordance with the animal care and use guidelines and the protocol was approved by the Animal Welfare Committee (AWC) of Zhengzhou University School in China. No additional ethical approvals or consents were required.

### 2.18 Statistical analysis

Statistical analysis was performed using the SPSS 19.0 statistical software package (SPSS Inc). All experiments were carried out at least 3 times, and the results were presented as the mean ± standard deviation. The unpaired 2‐tailed *t* test was used for the comparison of parameters between groups. The Mann‐Whitney test was used for data that are not of normal distribution by SPSS software. The standard deviation (SD) value was calculated by Excel software. *P* values of <.05 were considered statistically significant.

## 3 RESULTS

### 3.1 HN1L is over‐expressed in breast cancer tissues and closely related to the invasion and metastasis of breast cancer

To investigate the role of HN1L in the development and progression of breast cancer, we decided to investigate the expression patterns of HN1L in TCGA breast cancer patient database with RNA sequencing (RNA‐seq) information. Patient survival and gene expression data of 1094 breast cancers were downloaded from the Cancer Genome Atlas (TCGA) database. Firstly, the expression of HN1L in 925 cases of breast cancer tissues was analysed and the results showed that HN1L was significantly up‐regulated in breast cancer tissues, which was positively correlated with M metastasis of breast cancer patients (Tables S1 and S2).

**FIGURE 1:**
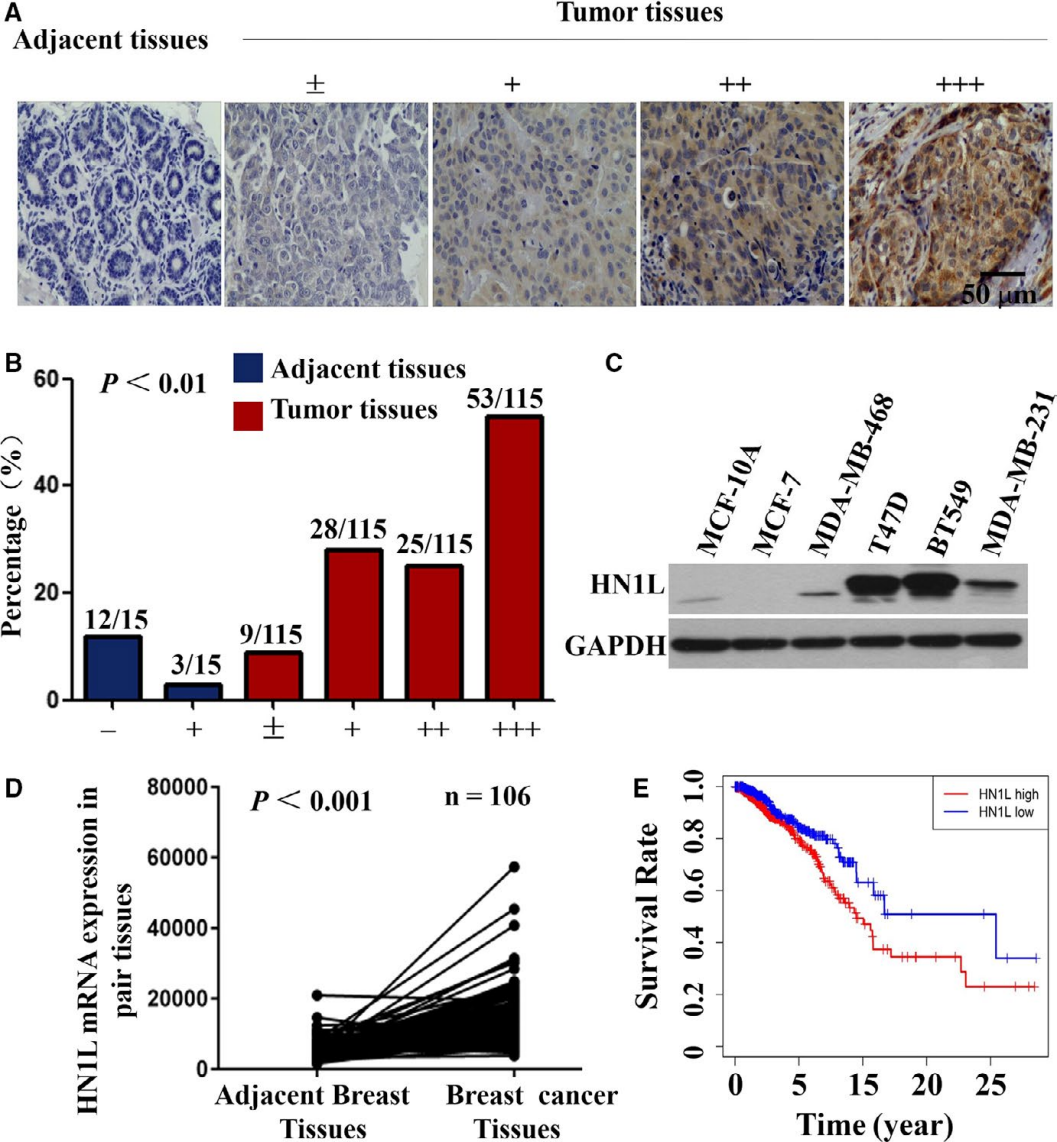
HN1L was over‐expressed in breast cancer tissues. (A) IHC staining of human breast cancer tissues arrays using HN1L‐specific antibodies. According to staining intensity, samples were classified into five groups with increasing staining intensity from the weakest (±, group 1) to the strongest (+++, group 4). (B) Classification of tumour samples according to the staining intensity of HN1L. Mann‐Whitney Test was used to evaluate the statistical significance of differences between groups. (C) Detection of HN1L Expression in Breast Cancer Cells by Western Blot assay. (D) Quantitative real‐time PCR analysed HN1L expression in breast cancer tissues and adjacent tissues in TCGA database. (E) Higher HN1L expression portends significantly poorer overall survival in TCGA breast cancer patients

Next, the expression of HN1L was evaluated using immunohistochemistry (IHC) staining from 15 adjacent breast tissues and 115 breast cancer tissues collected in Henan Cancer Hospital from 2013‐2014. Samples were divided into four groups with increasing intensity from the weakest (±, group 1) to the strongest (+++, group 4) (Figure 1A). Statistical analysis showed that the positive expression rate of HN1L was 71.3% in breast cancer tissues and 20% in adjacent normal tissues (Table S3). HN1L was over‐expressed in breast cancer tissues compared with adjacent normal tissues (Figure 1B). Meanwhile, HN1L was also expressed in the highly aggressive breast cancer cell lines MDA‐MB‐231, BT549 and T47D (Figure 1C). These results were consistent with the results of 106 pairs of breast cancer and paracancerous tissues in the TCGA database (Figure 1D). Besides, further analysis found that the expression of HN1L was closely related to Androgen receptor (AR), Ki‐67, Clinical nodal stage and TNM stage (Table S4, *P* < .05). Taken together, these findings suggested that the expression of HN1L was up‐regulated in breast cancer tissues, which was closely related to the invasion and metastasis of breast cancer. The survival analysis results in the TCGA data show that breast cancer patients with high HN1L expression have a poor prognosis (Figure 1E).

### 3.2 Knockdown of HN1L inhibits the proliferation of breast cancer cells

**FIGURE 2:**
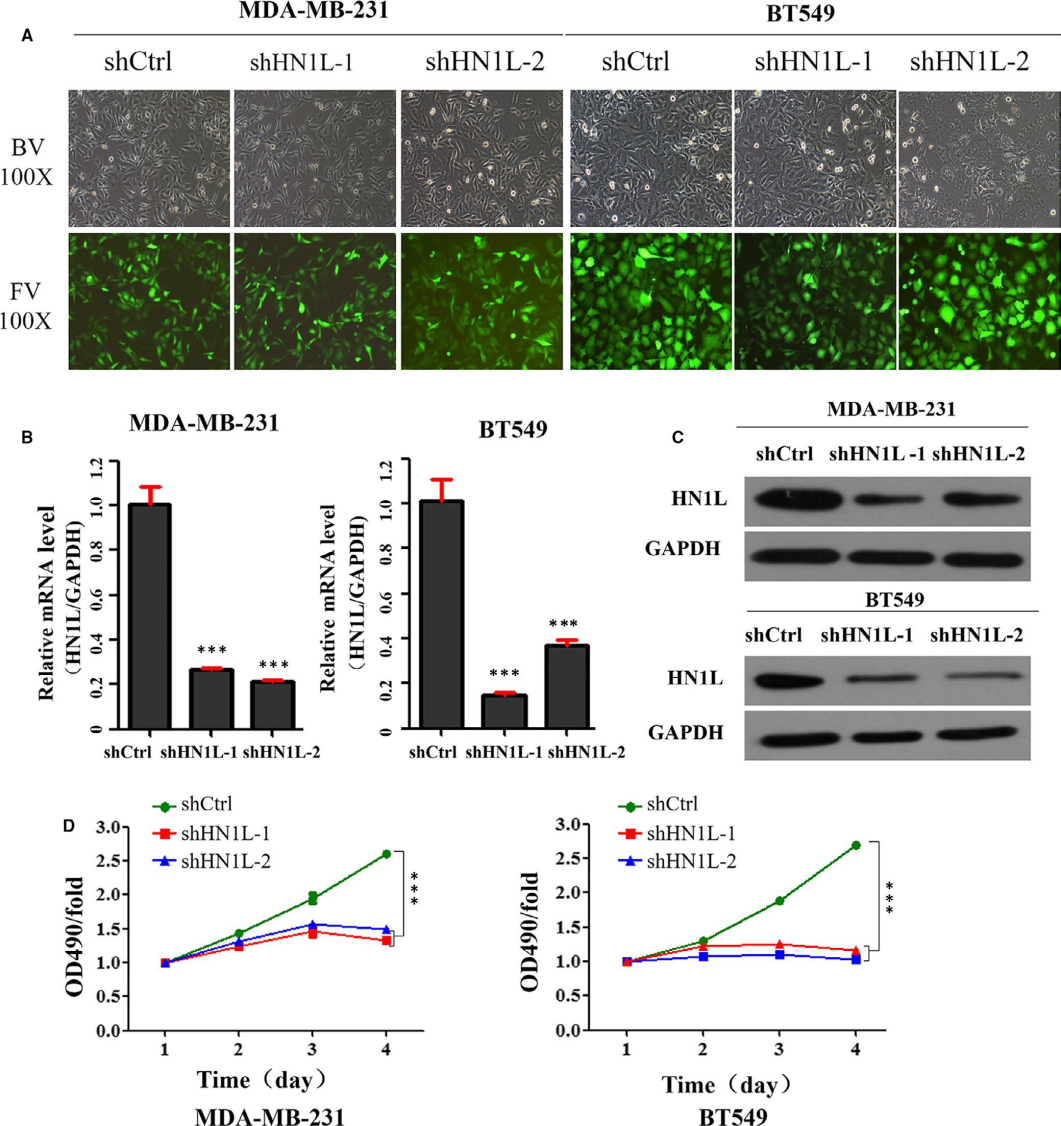
Knockdown of HN1L inhibits breast cancer cells growth. (A) Morphology of breast cancer cells after silencing HN1L. (B and C) Knockdown efficiency of shRNA targeting HN1L. Cells were infected with lentivirus containing shRNA for 72 h, RNA was collected and knockdown efficiency was determined by Real‐time PCR (***P < .001) (B), protein was collected and examined by western blot (C). (D) Effect of HN1L silencing on the viability of breast cancer cells MDA‐MB‐231 and BT549. Cells were knocked down HN1L for 72 h and cell viability was assessed by the MTT assay

In order to determine the function of HN1L in the invasion and metastasis of breast cancer, RNA interference technique was used to knock out the expression of HN1L. We selected highly invasive MDA‐MB‐231 and BT549 cells with different HN1L expression levels for the following study. Lentivirus infection assay was used to knock down HN1L expression in both MDA‐MB‐231 and BT‐549 cells in vitro (Figure 2A). Knockdown efficiency of shRNA targeting HN1L was detected by Q‐PCR and Western Blot (Figure 2B,C). In a cell growth assay, we found that the ability of cell proliferation was drastically decreased after HN1L knockdown in both cell lines (Figure 2D).

### 3.3 Knockdown of HN1L inhibits the invasion and metastasis of breast cancer cells

**FIGURE 3:**
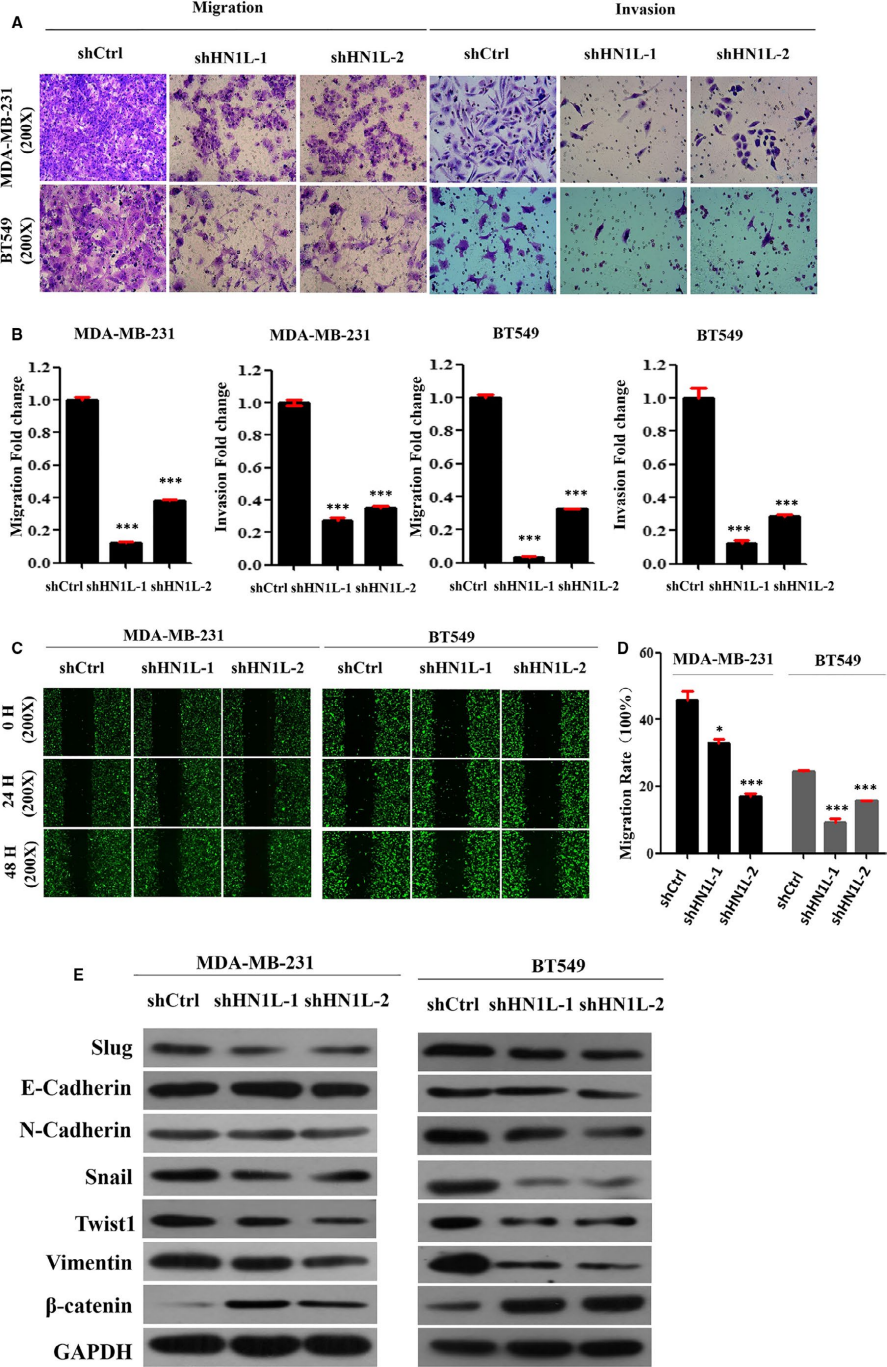
Knockdown of HN1L inhibits the invasion and metastasis of breast cancer cells. (A and B) Transwell invasion analysis showing the invasion ability of indicated cells with HN1L knockdown (**P < .01). (C and D) Wound healing assay showing the migration ability of MDA‐MB‐231 and BT549 with HN1L knockdown (**P < .01). (E) Western blot assay was applied to assess the expression of EMT marker proteins in the HN1L‐silencing cell lines and its control

To further assess the role of HN1L on the invasion and metastasis of breast cancer, stable HN1L knockdown cell line was generated and wound healing assay and transwell analysis were examined. As shown in Figure 3A,B, knockdown of HN1L caused a significant reduction of invasion in BT459 and MDA‐MB‐231 cells compared with their control. Besides, the percentage of wound closure for Control MDA‐MB‐231 cells was 100%, whereas shHN1L cells showed 55% wound closure, and the average mobility of BT549 cells was reduced by 61% (Figure 3C,D), suggesting that knockdown of HN1L severely destroys the migration ability of MDA‐MB‐231 and BT549 cells. Furthermore, western blot assay was applied to assess the expression of seven EMT related proteins (E‐cadherin, β‐catenin, N‐cadherin, Snail, Twist1, Slug and vimentin)^22^ (Figure 3E). Knockdown of HN1L up‐regulated β‐catenin and down‐regulated N‐cadherin, Snail, Twist1, Slug and vimentin compared with control cells. These results showed that knockdown of HN1L significantly inhibited the migration and invasion of breast cancer cells perhaps by inhibiting the EMT process.

### 3.4 Knockdown of HN1L inhibits lung metastasis in vivo

**FIGURE 4:**
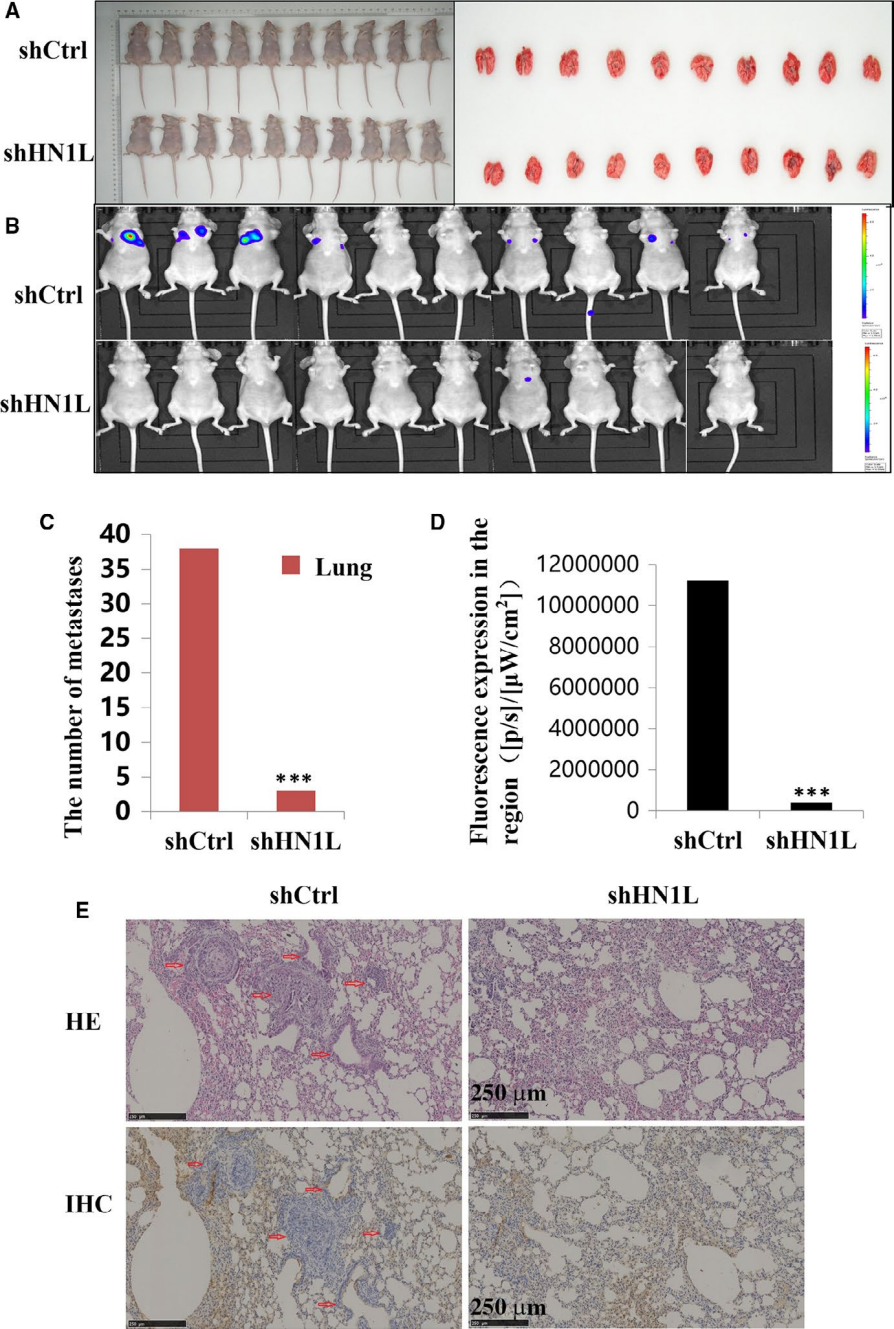
Knockdown of HN1L inhibits lung metastasis in vivo (n = 10). (A) Animal experiment animals and lungs pictures. (B) Small living animal imaging technology shows the expression of whole body fluorescence. (C) The metastatic nodules in the lungs were count following the orthotopic transplantat of Control and HN1L knockdowned cells (***P < .001). (D) Small living animal imaging technology data analysis show the expression of whole body fluorescence (***P < .001). (E) Immunohistochemical tests showed that HN1L gene silencing significantly inhibited lung metastasis

After demonstrating the inhibition efficacy of HN1L silencing in vitro, we further examined the effect of HN1L knockdown in vivo. Tumour growth and metastasis were monitored in NOD‐SCID mice implanted with control and HN1L knockdown MDA‐MB‐231 cells. The tumour weight showed no statistic difference between Control and HN1L knockdown groups (Figure S1). However, knockdown of HN1L resulted in a 70% decrease of lung metastases (Figure 4A,C). Furthermore, small living animal imaging technology data analysis showed that the expression of whole body fluorescence was decreased in HN1L knockdown group (*P* < .05) (Figure 4B,D), suggesting that silencing HN1L reduced the metastasis rate of breast cancer. Finally, we further verified by immunohistochemical experiments. As shown in Figure 4E, the metastases in the shHN1L group were significantly less than those in the shControl group in HE staining and immunohistochemical images, consistent with the previous results (Figure 4E).

### 3.5 HN1L silencing inhibits the invasion and metastasis of breast cancer by regulating HMGB1

**FIGURE 5:**
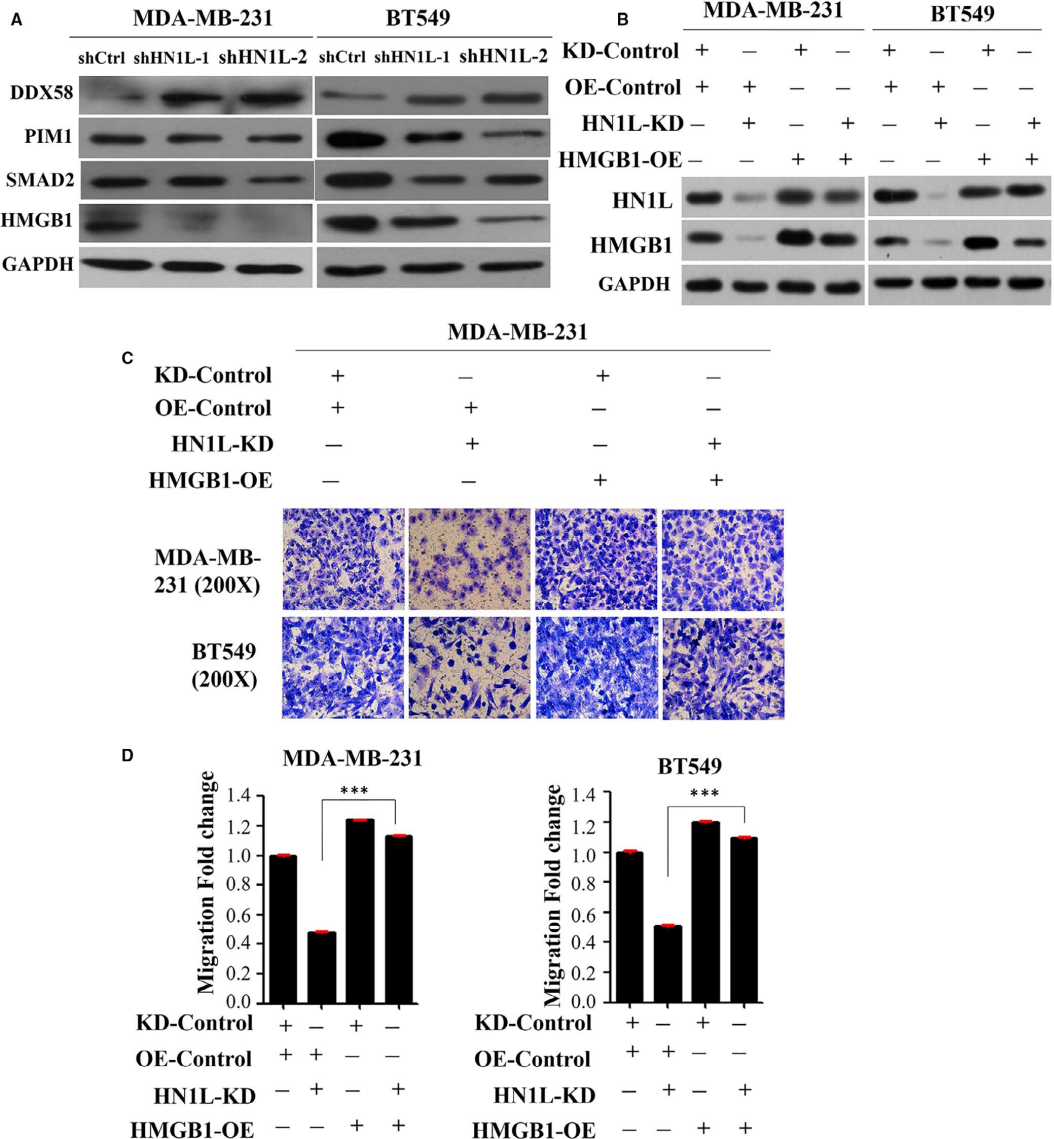
HN1L silencing inhibits invasion and Metastasis of Breast Cancer by regulating HMGB1. (A) Western blot assay was used to assess the expression of downstream proteins in the HN1L‐silencing cell line and control group. KD: Knockdown; OE: Over‐expression; (B) The efficiency of knockdown HSPA9 and overexpress HMGB1. (C and D) Transwell invasion analysis showing the invasion ability of breast cancer cell with HN1L knockdown and HMGB1 overexpress. (***P < .001)

To explore the target of HN1L, GeneChip primeview human analysis was used after silencing HN1L (Figure S2 and Table S5). Knockdown of HN1L led to a significant accumulation of DDX58, and a decrease of SMAD2, PIM1 and HMGB1 (Figure 5A). Recently, HMGB1 was reported to be over‐expressed in a number of cancers and involved in tumour invasion and metastasis.^23^, ^24^, ^25^, ^26^, ^27^ So we hypothesize that HN1L regulates the metastasis of breast cancer cell by HMGB1. To test this hypothesis, HMGB1 was over‐expressed in HN1L‐silencing cells. The efficiency of HN1L knockdown and HMGB1 over‐expression was confirmed by western blot assay (Figure 5B). HN1L knockdown significantly inhibited the migration ability of breast cancer cell, which was recovered by HMGB1 over‐expression (Figure 5C,D). These findings highlighted a pivotal role of HMGB1 in the process where HN1L‐silencing‐inhibited invasion and metastasis in breast cancer cell.

### 3.6 HMGB1 was likely regulated by the HN1L/HSPA9 interaction

**FIGURE 6:**
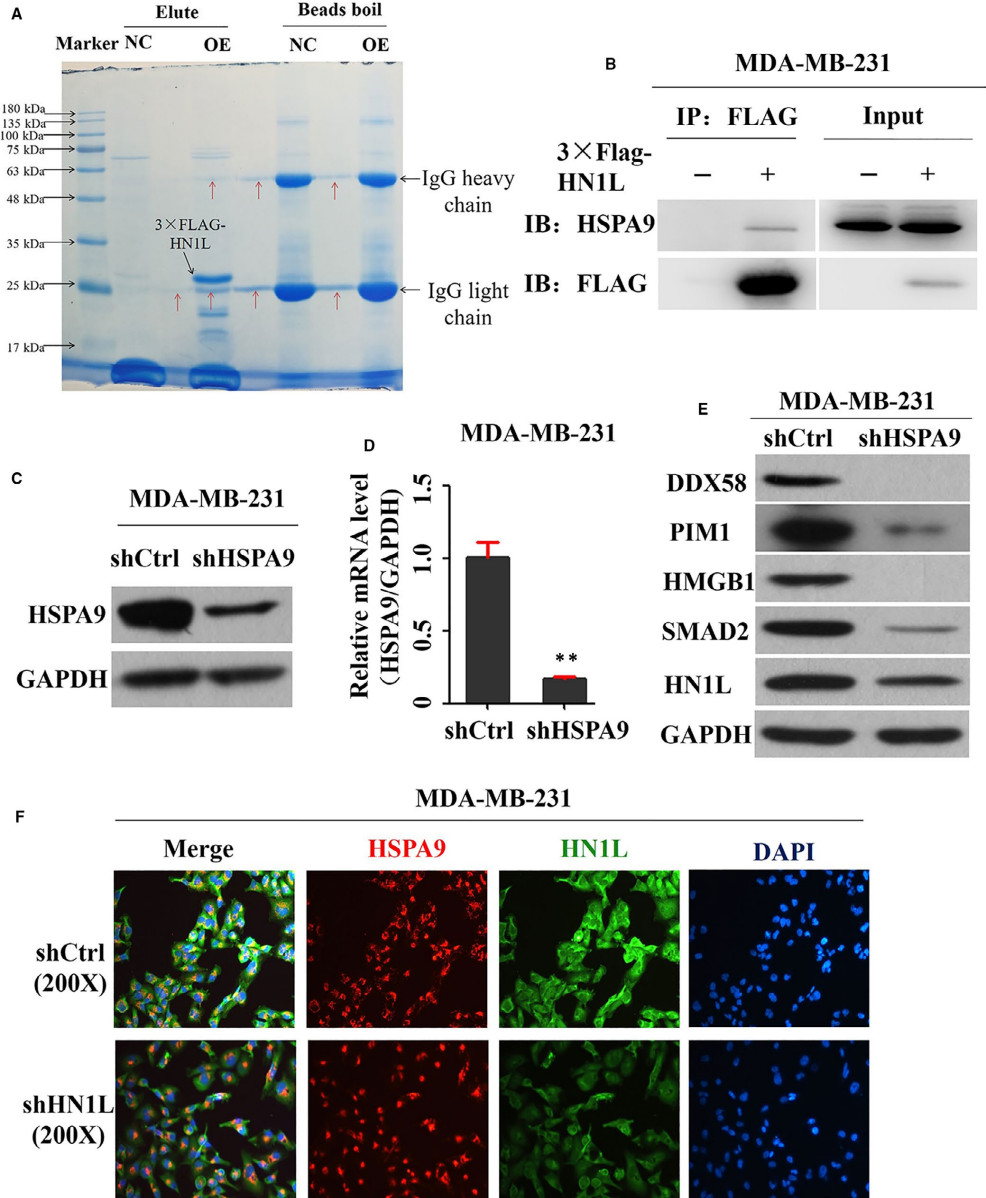
HMGB1 was regulated by the HN1L/HSPA9 interaction. (A and B) HSPA9 protein has a close interaction with HN1L. 3 × FLAG‐HN1L expressing cells and the control cells were purified using the anti‐FLAG magnetic beads and eluted by 150 μg/mL of 3 × FLAG peptide. Eluates were detected with HSPA9 and Flag antibodies for the interaction of HSPA9 with HNIL. WCL means whole cell lysis. (C and D) Knockdown efficiency of shRNA targeting HSPA9. Cells were infected with shRNA for 72 h, proteins and RNA were collected and knockdown efficiency was determined by western blot (C) and Q‐PCR (D) (**P < .01). (E) Western blot assay was applied to assess the expression of downstream proteins in the HSPA9‐silencing cell line and its control. (F) Immunofluorescence experiment showed that HN1L and HSPA9 were co‐expressed in breast cancer cells

To further investigate the molecular mechanism of HN1L in the invasion and metastasis of breast cancer, the protein interacting with HN1L was examined using LC/MS (Figure S3). Results suggested HSPA9 is one of the proteins that interact with HN1L, which was confirmed by the CO‐IP assay (Figure 6A,B). Next, the effect of HSPA9 on the metastasis was examined. HSPA9 was knockdown by a specific shRNA oligoes, named shHSPA9. The knockdown efficiency of shHSPA9 was confirmed by western blot (Figure 6C) and Q‐PCR assay (Figure 6D). Further results showed that knockdown of HSPA9 also led to the significant decrease of HMBG1 (Figure 6E). At the same time, immunofluorescence experiment showed that HN1L and HSPA9 were co‐expressed in breast cancer cells (Figure 6F). So we speculated that HMGB1 was likely regulated by the HN1L/HSPA9 interaction.

## 4 DISCUSSION

Nowdays, targeted therapy is one of the most important parts of breast cancer treatment and has shown good efficacy.^28^ For example, the molecular targeted drug trastuzumab has been used clinically and has shown good therapeutic effects.^29^, ^30^ Therefore, it is urgent to find new therapeutic target.

A previous report revealed the HN1L is a targetable breast cancer stem cell (BCSC) gene hinting its potential diagnostic and prognostic significance.^12^ In addition, the positive correlation of HN1L expression and Ki67 level in a large non‐small cell lung cancer samples further suggested the key role of HN1L in the regulation of cell growth.^11^ The results of TCGA database data analysis and IHC results also confirmed the over‐expression of HN1L in breast cancer. Furthermore, elevated HN1L was significantly associated with Ki67 level and lymph node metastasis in breast cancer patients (Table S4). Indeed, knockdown of HN1L significantly inhibited cell growth and decreased the tumorigenic potential of breast cancer cells, suggesting the essential oncogenic functions of HN1L in the development of breast cancer. Although the details of how HN1L works as an oncogene on cell proliferation are still unclear, the important role of HN1L in breast cancer function necessitates further study on its specific mechanisms.

Many studies have shown that the occurrence and development of breast cancer, including tumour metastasis, are complicated processes involving epithelial‐mesenchymal transition (EMT) and mesenchymal‐epithelial transition (MET).^31^, ^32^, ^33^ In addition, the homologous gene HN1 of HN1L contributes to the migration and invasion of breast cancer.^11^ Therefore, HN1L also likely to be involved in the regulation of invasion and metastasis of breast cancer. So the expression of EMT marker proteins was examined. Results showed that knockdown of HN1L up‐regulated β‐catenin and down‐regulated N‐cadherin, Snail, Twist1, Slug and vimentin compared with control cells. Moreover, knockdown of HN1L significantly inhibited the proliferation, invasion and metastasis in MDA‐MB‐231 and BT549 cells and lung metastasis in nude mice metastasis model of breast cancer. Thus, HN1L's involvement in the process of EMT of breast cancer cell is generally in line with HN1L's role as an oncogene.

Although HN1L expression has been reported to be significantly associated with shorter overall or recurrence‐free survival in triple‐negative breast cancer (TNBC) patients,^12^ the exact molecular mechanism remains unclear. Mechanistically, we examined the protein that interacted with HN1L. Among these proteins, we found a more interesting protein, HSPA9, which was related to proliferation, function maintenance and stress response in cancer cells. The human HSPA9 gene is located on chromosome 5q31.2 and encodes for one of the heat shock protein 70 family members, also known as mortalin/mthsp70/PBP74/GRP75.^34^ As a molecular chaperone, HSPA9 interacts with other proteins and functions in regulating cellular stress response, EMT,^35^ cell proliferation^36^ and apoptosis.^37^ HSPA9 was reported to be up‐regulated in many cancers.^38^, ^39^ Besides, the over‐expression of HSPA9 promoted invasion and metastasis of breast cancer and associated with histological grade, clinical stage and lymph node metastasis.^35^, ^40^ Emerging data have suggested that HMGB1 could promote tumour progression *via* promoting proliferation and invasiveness of cancer cells.^41^, ^42^ HMGB1 was highly expressed in many tumours, such as lung cancer,^41^ prostate cancer,^42^ gastric cancer,^27^ hepatocellular carcinoma,^43^ breast cancer,^26^ colorectal cancer^44^ and ovarian cancer.^45^ In addition, HMGB1 was highly expressed in renal cell carcinoma, and the expression level showed a positive correlation with cancer bearing, metastasis and clinical staging and grading.^46^, ^47^ As a multifunctional cytokine, HMGB1 plays a key role in tumour formation, metastasis and EMT.^48^, ^49^ In our study, Knockdown of HN1L led to a significant accumulation of DDX58, with a decrease of SMAD2, PIM1 and HMGB1. PIM‐1 can inhibit the growth of triple‐negative breast cancer cells,^50^ so the decrease of PIM1 expression caused by HN1L gene silencing may be one of the ways by which HN1L regulates the proliferation of breast cancer cells. Smad2 is the substrate of TGF‐ β receptor kinase and the key protein of TGF‐ β/ Smads signalling pathway.^51^ It plays an important role in cell proliferation, differentiation and apoptosis.^52^ Here, the decrease of SMAD2 expression after HN1L gene silencing may be the result of the effect of HN1L gene silencing on cell proliferation and apoptosis, but the detailed molecular mechanism of HN1L regulating breast cancer cell proliferation needs to be further studied. It is noteworthy that among the HN1L‐associated genes detected by ChIP‐seq, HMGB1 has been reported as a key regulator in the EMT process in mesothelial cells,^53^, ^54^ suggesting another possible mechanism of HN1L through regulation of HMGB1 expression. In our rescue experiment, over‐expression of HMGB1 in HN1L‐silenced breast cancer cells saved the migration ability of breast cancer cells, which suggests that HNIL affects the migration ability of breast cancer cells by regulating the expression of HMGB1. Besides, HMGB1 was also reported as a senescence marker.^55^, ^56^ Meanwhile, recent studies show that depleted cells of the mitochondrial chaperone HSPA9 induced cell senescence.^56^ Considering that HSPA9 is involved in cell senescence and HMGB1 is one of the markers of cell senescence, we hypothesize that HNIL may interact with HSPA9 and participate in breast cancer cell invasion and metastasis mediated by HMGB1.

To test this hypothesis, CO‐IP and immunofluorescence assays were first used to find the interaction of HN1L and HSPA9. Results showed that HN1L and HSPA9 were co‐expressed and interacted in breast cancer cells which were in line with our hypothesis. Consistent with knockdown of HN1L, HSPA9 knockdown also caused a significant decrease in HMGB1 protein expression. These studies suggest that HN1L/HSPA9‐HMGB1 perhaps play an important role in the migration and invasion of breast cancer. However, the detailed mechanism of the interaction between HN1L and HSPA9 affecting the expression of HMGB1 still needs further experimental studies.

In summary, our study proves that HN1L silencing can inhibit the migration and invasion of breast cancer cells in vitro, and lung metastasis in vivo. Although our study offers new insight into the mechanisms of HN1L in breast cancer metastasis, the specific regulatory mechanisms of the interactions among HN1L, HSPA9 and HMGB1 need further study. Taken together, HN1L may provide promising diagnostic and therapeutic options for breast cancer patients in the future.

## CONFLICT OF INTEREST

No potential competing interests are disclosed.

## AUTHOR CONTRIBUTIONS

**Dechuang Jiao:** Data curation (equal); formal analysis (equal); investigation (equal). **Jingyang Zhang:** Methodology (equal); project administration (equal). **Ping Chen:** Project administration (equal); resources (equal); writing‐review & editing (equal). **Xuhui Guo:** Data curation (equal); software (equal). **Jianghua Qiao:** Supervision (equal); writing‐original draft (equal). **Jiujun Zhu:** Data curation (equal); validation (equal). **Lina Wang:** Visualization (equal); writing‐original draft (equal). **Zhenduo Lu:** Writing‐review & editing (equal). **Zhenzhen Liu:** Conceptualization (equal); funding acquisition (equal).

## Supporting information

Fig S1

Fig S2

Fig S3

Table S1‐4

Table S5

Legends S1

## Data Availability

Data sharing is not applicable to this article as no new data were created or analyzed in this study.
