# Supplementary figures and images for: HN1L promotes migration and invasion of breast cancer by up‐regulating the expression of HMGB1
Source: J Cell Mol Med. 2020 Nov 16;25(1):397–410. doi: 10.1111/jcmm.16090 (PMC7810958; doi:10.1111/jcmm.16090)

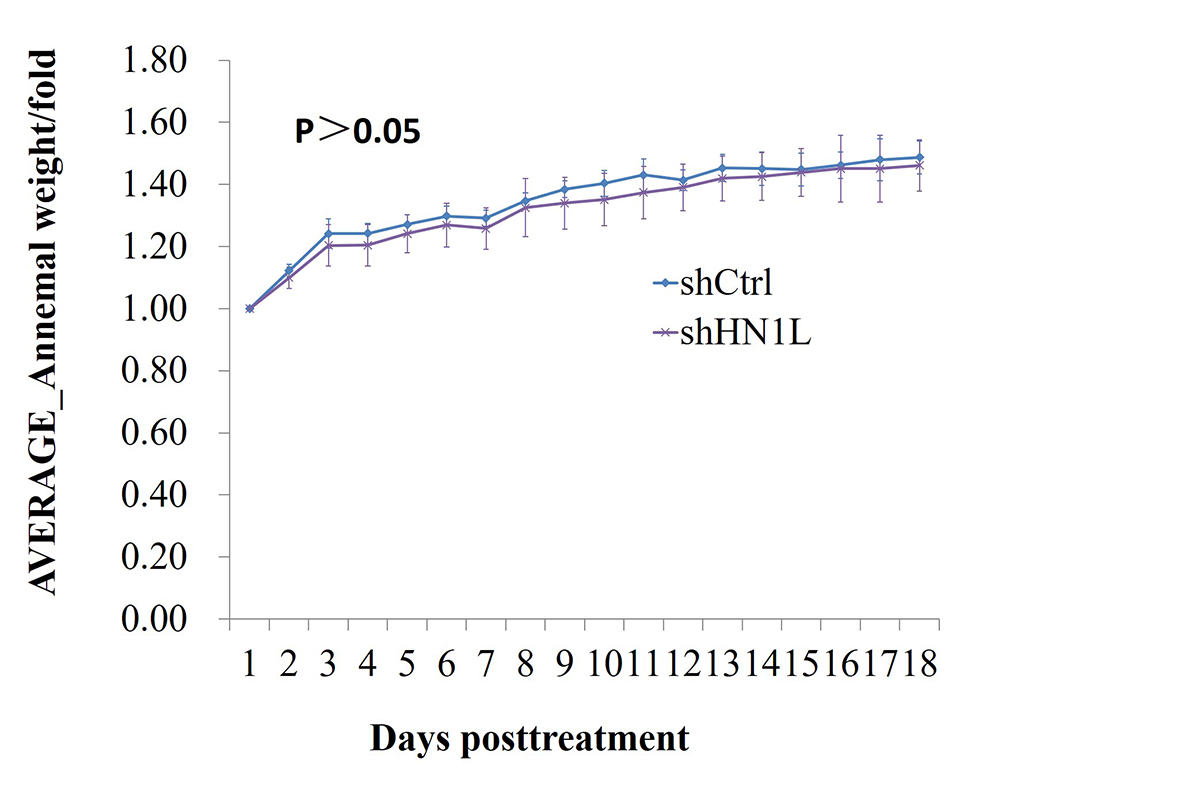

Supplement: Supplementary file 1 — Fig S1 [file JCMM-25-397-s001.tif]

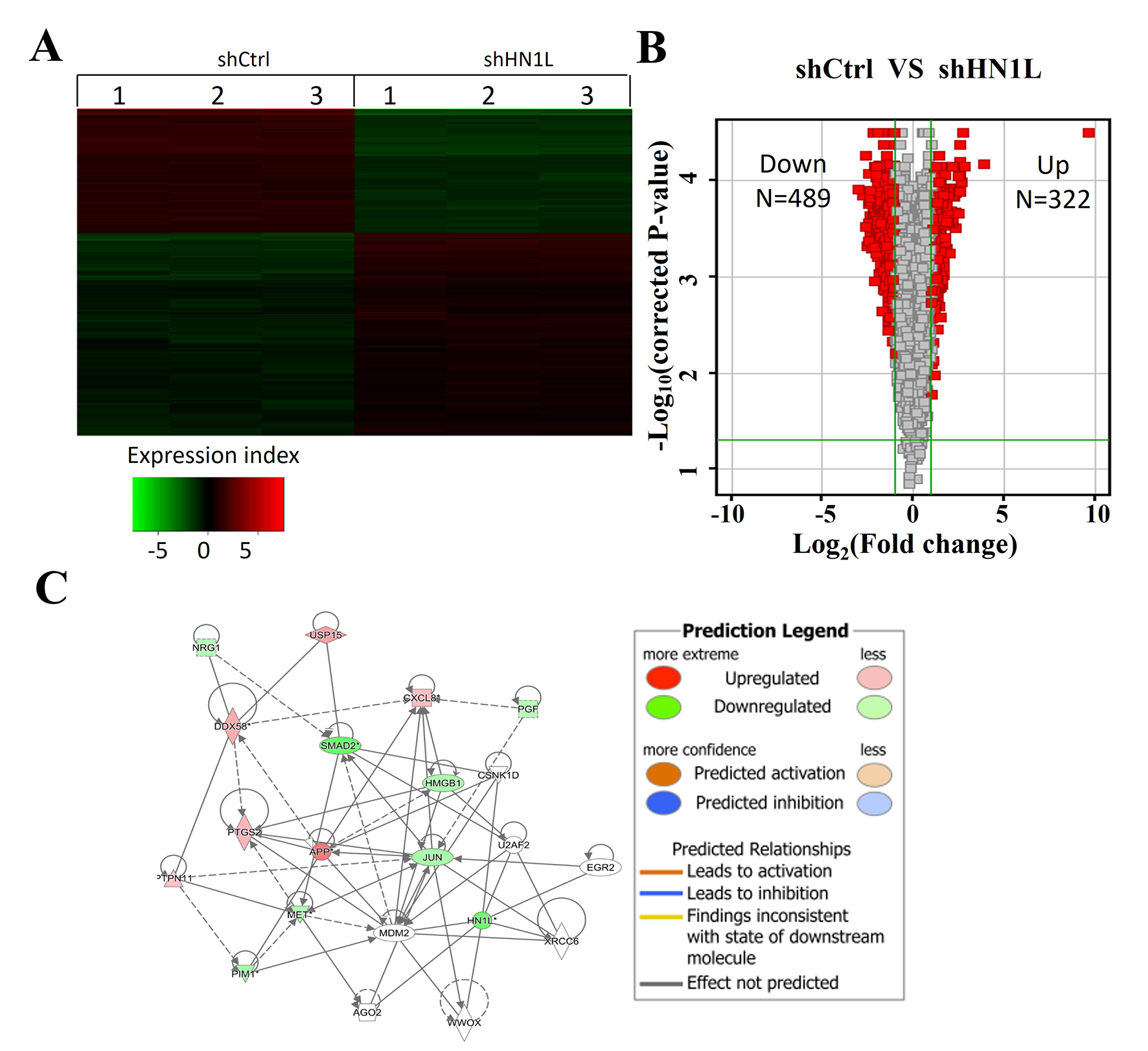

Supplement: Supplementary file 2 — Fig S2 [file JCMM-25-397-s002.tif]

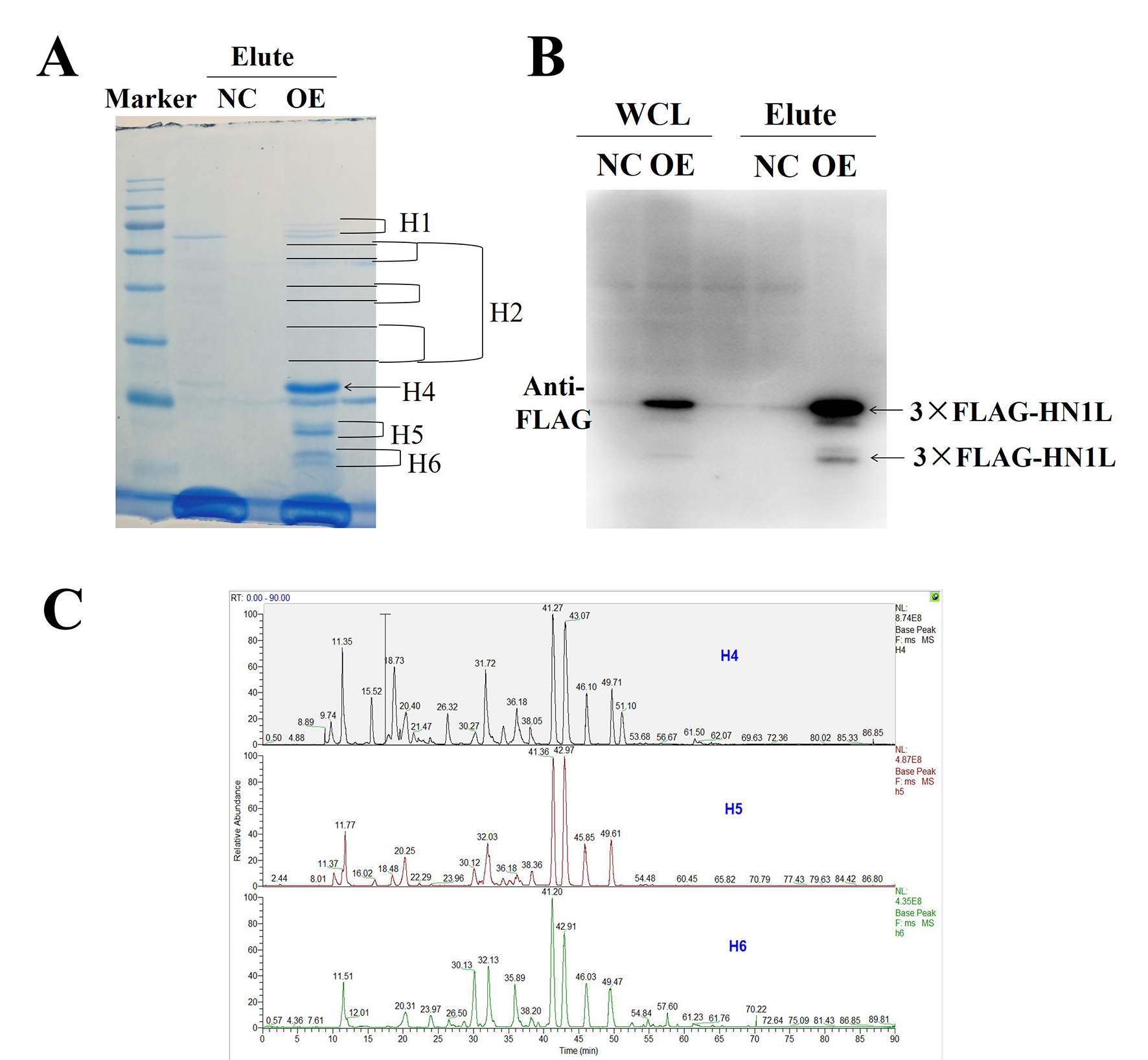

Supplement: Supplementary file 3 — Fig S3 [file JCMM-25-397-s003.tif]
