# Supplementary material for: HN1L promotes migration and invasion of breast cancer by up‐regulating the expression of HMGB1
Source: J Cell Mol Med. 2020 Nov 16;25(1):397–410. doi: 10.1111/jcmm.16090 (PMC7810958; doi:10.1111/jcmm.16090)
Supplement: Supplementary file 4 — Table S1‐4 [file JCMM-25-397-s004.docx]

**Table Legends**

**Table S1. High expression of HN1L is related with M metastasis of breast cancer**

|  | | HN1L | | Overall  No. | *P* |
| --- | --- | --- | --- | --- | --- |
|  |  | Low expression | High expression |  |  |
| M metastasis | M0 | 439 | 464 | 903 | 0.048 |
|  | M1 | 6 | 16 | 22 |  |
| Total* | | 445 | 480 | 925 |  |

*Among the 1094 breast cancer patients, 925 cases had accurate M metastasis data.

**Table S2. Correlation between the expression of HN1L in cancer tissue and clinical data**

|  |  | HN1L expression | M metastasis |
| --- | --- | --- | --- |
| HN1L expression | correlation coefficient | 1.000 | 0.065 |
|  | Sig. |  | 0.048 |
| M  metastasis | correlation coefficient | 0.056 | 1.000 |
|  | Sig. | 0.048 |  |

**Table S3. Expression of HN1L in breast cancer and adjacent mammary tissues**

| Characteristics | Total | HN1L | | *P* |
| --- | --- | --- | --- | --- |
|  |  | Low | High |  |
| Adjacent | 15 | 12(80%) | 3(20%) | <0.001 |
| Cancer | 115 | 33(28.7%) | 82(71.3%) |  |

**Table S4. Association of HN1L expression with clinicopathologic parameters of breast cancer patients**

| Characteristics | | Total | HN1L | | *P* |
| --- | --- | --- | --- | --- | --- |
|  |  |  | Low | High |  |
| Overall | | 115 | 33 | 82 | - |
| ER | Negative | 44 | 12 | 32 | 0.791 |
|  | Positive | 71 | 21 | 50 |  |
| PR | Negative | 48 | 14 | 34 | 0.925 |
|  | Positive | 67 | 19 | 48 |  |
| AR | Negative | 24 | 12 | 12 | 0.009 |
|  | Positive | 91 | 21 | 70 |  |
| HER-2 status | Negative | 73 | 24 | 49 | 0.191 |
|  | Positive | 42 | 9 | 33 |  |
| Ki-67 | Low | 40 | 4 | 36 | 0.003 |
|  | High | 75 | 29 | 46 |  |
| Clinical nodal stage(N) | N0 | 55 | 14 | 41 | 0.024 |
|  | N1 | 35 | 16 | 19 |  |
|  | N2 | 17 | 3 | 14 |  |
|  | N3 | 8 | 0 | 8 |  |
| TNM stage | I | 14 | 2 | 15 | 0.037 |
|  | II | 62 | 26 | 44 |  |
|  | III+IV | 35 | 5 | 23 |  |
| Tumor (T) stage | T1 | 23 | 3 | 20 | 0.231 |
|  | T2 | 79 | 27 | 52 |  |
|  | T3 | 8 | 2 | 6 |  |
|  | T4 | 5 | 1 | 4 |  |
