# Supplementary material for: HN1L promotes migration and invasion of breast cancer by up‐regulating the expression of HMGB1
Source: J Cell Mol Med. 2020 Nov 16;25(1):397–410. doi: 10.1111/jcmm.16090 (PMC7810958; doi:10.1111/jcmm.16090)
Supplement: Supplementary file 6 — Legends S1 [file JCMM-25-397-s006.docx]

**Table S1.** High expression of HN1L is related with M metastasis of breast cancer. Patient survival and gene expression data of 1094 breast cancers were downloaded from the Cancer Genome Atlas (TCGA) database. Among the 1094 breast cancer patients, 925 cases had accurate M metastasis data.

**Table S2**. Correlation between the expression of HN1L in cancer tissue and clinical data. HN1L was positively correlated with M metastasis of breast cancer patients.

**Table S3**. Expression of HN1L in breast cancer and adjacent mammary tissues. Statistical analysis showed that the positive expression rate of HN1L was 71.3% in breast cancer tissues and 20% in adjacent tissues.

**Table S4**. Association of HN1L expression with clinicopathologic parameters of breast cancer patients. The expression of HN1L was closely related to Androgen receptor (AR), Ki-67, Nodal stage and TNM stage.

**Table S5**. The result of the GeneChip PrimeView chip： The list of up-regulated and down-regulated genes.

**Fig S1 Animal weight change chart in animal experiment.** The tumour weight showed no statistic difference between Control and HN1L knockdown groups. 2×10^7^ MDA-MB-231 cells were subcutaneously injected into the tail vein of immunocompromised NOD/SCID mice (n=20). The weight of animal was monitored every 3 days.

**Fig S2 GeneChip primeview human analysis A:** The cluster diagram shows the aggregation of all samples and differential genes at the expression level. Red indicates that the signal value of the gene is relatively up-regulated. Green indicates that the signal value of the gene is relatively down-regulated, black indicates that the signal value of the gene is moderate, and grey indicates that the signal value of the gene is not detected. **B:** The volcano map shows the distribution of differential genes between the experimental group and the control group. Red indicates all genes with a multiple of difference greater than 2 and a significance level less than 0.05. **C:** Gene network diagram: In the network diagram, genes, proteins, chemicals, etc. are represented in different shapes; the colour of the molecule is shown in the legend.

**Figure S3 Screening of HN1L interacting complexes by Co-IP combined with LC-MS/MS. (NC: Control group; OE: 3 × FLAG-HN1L over-expressed group)** A: Co-IP samples were separated by SDS-PAGE, and after elution, cut the difference bar: H4 should be the main 3×FLAG-HN1L protein. The band of H5 and H6 should be 3×FLAG-HN1L (spliced body). B: Co-IP sample western blot quality control: The results of IP samples show that 3×FLAG-HN1L can be effectively enriched. C: MS map: The bands of H5 and H6 in figure B are very similar to the peaks of H4, H5 and H6 shown by MS, suggesting that both H5 and H6 bands are 3 × FLAG-HN1L proteins.
